# *catena*-Poly[[diaqua­bis­[2-(3-oxo-1,3-di­hydro-2-benzofuran-1-yl)acetato-κ*O*]cobalt(II)]-μ-1,2-bis­(pyridin-4-yl)ethane-κ^2^ *N*:*N*′]

**DOI:** 10.1107/S1600536812044339

**Published:** 2012-10-31

**Authors:** Changyan Sun, Yang Li, Wenjun Li, Bin Dong

**Affiliations:** a School of Chemistry and Biological Engineering, University of Science and Technology Beijing, Beijing 100083, People’s Republic of China

## Abstract

In the title complex, [Co(C_10_H_7_O_4_)_2_(C_12_H_12_N_2_)(H_2_O)_2_]_*n*_, the Co^II^ ion is located on a crystallographic centre of symmetry and is six-coordinated by two N atoms from two 1,2-bis­(4-pyrid­yl)ethane ligands, two carboxylate O atoms from two 1,3-dihydro-3-oxo-1-isobenzofuran­acetate ligands and two terminal water ligands. The 1,2-bis(4-pyrid­yl)ethane ligands act as bidentate ligands, and bridge the Co^II^ ions into infinite chains extending parallel to [010]. In these chains, there are intra-mol­ecular O—H⋯O hydrogen bonding between the coordination water mol­ecules and carboxyl­ate groups. Inter-mol­ecular O—H⋯O hydrogen bonding between the adjacent chains and π⋯π stacking inter­actions result in the formation of a three-dimensional supra­molecular network.

## Related literature

For *in situ* ligand reactions, see: Zhang *et al.* (2005 ▶); Chen *et al.* (2007 ▶); Zhao *et al.* (2008 ▶).
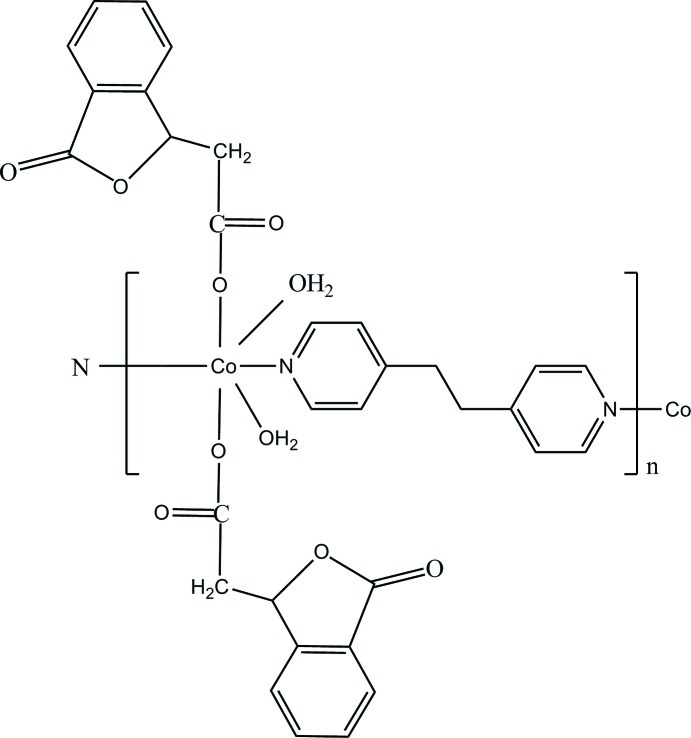


## Experimental

### 

#### Crystal data

[Co(C_10_H_7_O_4_)_2_(C_12_H_12_N_2_)(H_2_O)_2_]
*M* *_r_* = 661.51
Triclinic, 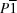

*a* = 5.4599 (12) Å
*b* = 10.374 (2) Å
*c* = 13.617 (3) Å
α = 93.912 (4)°
β = 99.409 (4)°
γ = 97.651 (4)°
*V* = 750.9 (3) Å^3^
*Z* = 1
Mo *K*α radiation
μ = 0.63 mm^−1^
*T* = 293 K
0.20 × 0.18 × 0.10 mm

#### Data collection

Bruker SMART CCD area-detector diffractometer
Absorption correction: multi-scan (*SADABS*; Bruker, 1997 ▶) *T*_min_ = 0.749, *T*_max_ = 1.000
4258 measured reflections
3027 independent reflections
2306 reflections with *I* > 2σ(*I*)
*R*_int_ = 0.026

#### Refinement

*R*[*F* ^2^ > 2σ(*F* ^2^)] = 0.055
*wR*(*F* ^2^) = 0.133
*S* = 1.05
3027 reflections
213 parameters
H atoms treated by a mixture of independent and constrained refinement
Δρ_max_ = 0.73 e Å^−3^
Δρ_min_ = −0.61 e Å^−3^

### 

Data collection: *SMART* (Bruker, 1997 ▶); cell refinement: *SAINT* (Bruker, 1997 ▶); data reduction: *SAINT*; program(s) used to solve structure: *SHELXS97* (Sheldrick, 2008 ▶); program(s) used to refine structure: *SHELXL97* (Sheldrick, 2008 ▶); molecular graphics: *SHELXTL* (Sheldrick, 2008 ▶); software used to prepare material for publication: *SHELXTL*.

## Supplementary Material

Crystal structure: contains datablock(s) global, I. DOI: 10.1107/S1600536812044339/hg5263sup1.cif

Supplementary material file. DOI: 10.1107/S1600536812044339/hg5263Isup2.cdx

Structure factors: contains datablock(s) I. DOI: 10.1107/S1600536812044339/hg5263Isup3.hkl

Additional supplementary materials:  crystallographic information; 3D view; checkCIF report

## Figures and Tables

**Table 1:** Hydrogen-bond geometry (Å, °)

*D*—H⋯*A*	*D*—H	H⋯*A*	*D*⋯*A*	*D*—H⋯*A*
O5—H5*B*⋯O2	0.90 (5)	1.72 (5)	2.603 (4)	168 (4)
O5—H5*B*⋯O1	0.90 (5)	2.57 (4)	2.981 (3)	109 (3)
O5—H5*A*⋯O1^i^	0.76 (4)	1.99 (4)	2.752 (3)	172 (4)

Symmetry code: (i) 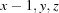.

**Fig. 1.**
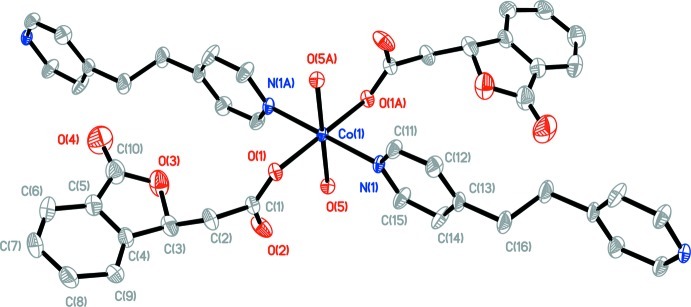
The coordination environment of CoII ion in the title complex (the symmetry code for A: -x + 1, -y + 1, -z + 1).

**Fig. 2.**
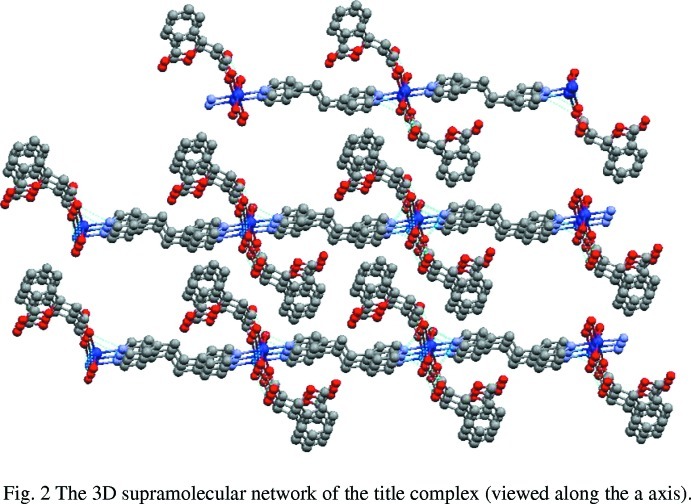
The three-dimensional supramolecular network of the title complex (viewed along the a axis).

## supplementary crystallographic information

### Comment

In situ ligand reactions have been extensively used to construct new organic ligands and coordination polymers, with novel structures and potential applications, especially those that could not be obtained in direct preparation from the ligands. Furthermore, they are helpful to study reaction mechanism (Zhang *et al.*, 2005; Chen *et al.*, 2007; Zhao *et al.*, 2008). Herein we report the synthesis and structure of the title complex from in situ hydroxylation and esterification of ethylene group. In the title complex (Fig. 1), each Co^II^ ion is located on a crystallographic centre of symmetry and coordinated to four oxygen atoms and two nitrogen atoms, and the coordination geometry can be described as a distorted octahedron. 1,3-Dihydro-3-oxo-1 -isobenzofuranacetate ligand adopts a monodentate mode, coordinating to Co^II^ ion with a carboxylate oxygen atom. While 1,2-bis(4-pyridyl)ethane ligands bridge Co^II^ ions into one-dimensional infinite chains. In these chains, intra-molecular O—H···O hydrogen bonding are observed between the carboxylate oxygen atoms and the coordination water molecules (O(2)···O(5) 2.603 (4) Å and O(1)···O(5) 2.981 (3) Å). Between the adjacent chains, there are inter-molecular O—H···O hydrogen bonding (O(1)···O(5) 2.752 (3) Å), which connects the chains into a three-dimensional supramolecular network (Table 1 and Fig. 2). 1,3-Dihydro-3-oxo-1-isobenzofuranacetate ligands are parallel and the face-to-face distance is 3.88 Å, indicating the existence of the weak π···π stacking interactions, which brings further stability for the structure.

### Experimental

A mixture of *o*-carboxycinnamic acid (0.02 g, 0.1 mmol), CoCl_2_.6H_2_O (0.024 g, 0.1 mmol), 1,2-bis(4-pyridyl)ethane (0.018 g, 0.1 mmol), and deionized water(5 ml) was sealed in a Teflon-lined stainless vessel(25 ml), and heated at 120 °C for 72 h, then cooled slowly to room temperature. There was no solid obtained. The filtration was left to stand in air. After two weeks, red block single crystals were obtained. Yield: 0.015 g (22.7%).

### Refinement

The water H atoms were located in the Fourier difference map and refined with isotropic coordinates. The carbon H atoms were included in the refinement in the riding model approximation, with C–H bond distance 0.93–0.98 Å and *U*(H)set to 1.2*U*_eq_(C).

### Crystal data

**Table:**

[Co(C_10_H_7_O_4_)_2_(C_12_H_12_N_2_)(H_2_O)_2_]	*Z* = 1
*M**_r_* = 661.51	*F*(000) = 343
Triclinic, *P*1	*D*_x_ = 1.463 Mg m^−^^3^
Hall symbol: -P 1	Mo *K*α radiation, λ = 0.71073 Å
*a* = 5.4599 (12) Å	Cell parameters from 1338 reflections
*b* = 10.374 (2) Å	θ = 1.5–26.4°
*c* = 13.617 (3) Å	µ = 0.63 mm^−^^1^
α = 93.912 (4)°	*T* = 293 K
β = 99.409 (4)°	Block, red
γ = 97.651 (4)°	0.20 × 0.18 × 0.10 mm
*V* = 750.9 (3) Å^3^	

### Data collection

**Table:**

Bruker SMART CCD area-detector diffractometer	3027 independent reflections
Radiation source: fine-focus sealed tube	2306 reflections with *I* > 2σ(*I*)
Graphite monochromator	*R*_int_ = 0.026
phi and ω scans	θ_max_ = 26.4°, θ_min_ = 1.5°
Absorption correction: multi-scan (*SADABS*; Bruker, 1997)	*h* = −4→6
*T*_min_ = 0.749, *T*_max_ = 1.000	*k* = −12→12
4258 measured reflections	*l* = −16→17

### Refinement

**Table:**

Refinement on *F*^2^	Primary atom site location: structure-invariant direct methods
Least-squares matrix: full	Secondary atom site location: difference Fourier map
*R*[*F*^2^ > 2σ(*F*^2^)] = 0.055	Hydrogen site location: inferred from neighbouring sites
*wR*(*F*^2^) = 0.133	H atoms treated by a mixture of independent and constrained refinement
*S* = 1.05	*w* = 1/[σ^2^(*F*_o_^2^) + (0.051*P*)^2^ + 0.6286*P*] where *P* = (*F*_o_^2^ + 2*F*_c_^2^)/3
3027 reflections	(Δ/σ)_max_ < 0.001
213 parameters	Δρ_max_ = 0.73 e Å^−^^3^
0 restraints	Δρ_min_ = −0.61 e Å^−^^3^

### Special details

**Table:**

Geometry. All e.s.d.'s (except the e.s.d. in the dihedral angle between two l.s. planes) are estimated using the full covariance matrix. The cell e.s.d.'s are taken into account individually in the estimation of e.s.d.'s in distances, angles and torsion angles; correlations between e.s.d.'s in cell parameters are only used when they are defined by crystal symmetry. An approximate (isotropic) treatment of cell e.s.d.'s is used for estimating e.s.d.'s involving l.s. planes.
Refinement. Refinement of *F*^2^ against ALL reflections. The weighted *R*-factor *wR* and goodness of fit *S* are based on *F*^2^, conventional *R*-factors *R* are based on *F*, with *F* set to zero for negative *F*^2^. The threshold expression of *F*^2^ > σ(*F*^2^) is used only for calculating *R*-factors(gt) *etc*. and is not relevant to the choice of reflections for refinement. *R*-factors based on *F*^2^ are statistically about twice as large as those based on *F*, and *R*- factors based on ALL data will be even larger.

### Fractional atomic coordinates and isotropic or equivalent isotropic displacement parameters (Å^2^)

**Table:**

	*x*	*y*	*z*	*U*_iso_*/*U*_eq_	
Co1	0.5000	0.5000	0.5000	0.0231 (2)	
N1	0.5225 (5)	0.4729 (3)	0.34064 (18)	0.0312 (7)	
O1	0.6967 (4)	0.3420 (2)	0.52914 (16)	0.0326 (6)	
O2	0.3880 (5)	0.1819 (3)	0.5357 (3)	0.0613 (9)	
O3	0.7376 (7)	0.1884 (4)	0.7644 (2)	0.0817 (12)	
O4	0.9063 (9)	0.2427 (5)	0.9246 (3)	0.1123 (17)	
C1	0.6125 (7)	0.2293 (4)	0.5499 (3)	0.0358 (9)	
C2	0.8056 (8)	0.1465 (4)	0.5951 (3)	0.0498 (11)	
H2A	0.8187	0.0778	0.5451	0.060*	
H2B	0.9680	0.2009	0.6124	0.060*	
C3	0.7443 (8)	0.0860 (5)	0.6858 (3)	0.0566 (12)	
H3	0.5815	0.0296	0.6696	0.068*	
C4	0.9426 (8)	0.0100 (5)	0.7330 (3)	0.0523 (11)	
C5	1.0204 (9)	0.0604 (5)	0.8312 (3)	0.0574 (12)	
C6	1.1928 (11)	0.0029 (6)	0.8936 (4)	0.0823 (17)	
H6	1.2403	0.0335	0.9607	0.099*	
C7	1.2914 (11)	−0.0991 (6)	0.8550 (4)	0.0839 (18)	
H7	1.4078	−0.1382	0.8961	0.101*	
C8	1.2209 (10)	−0.1446 (5)	0.7563 (4)	0.0710 (15)	
H8	1.2932	−0.2131	0.7311	0.085*	
C9	1.0467 (9)	−0.0916 (5)	0.6938 (4)	0.0640 (13)	
H9	0.9998	−0.1231	0.6268	0.077*	
C10	0.8928 (11)	0.1723 (6)	0.8499 (4)	0.0753 (16)	
C11	0.7230 (7)	0.5193 (4)	0.3025 (3)	0.0454 (10)	
H11	0.8650	0.5601	0.3464	0.054*	
C12	0.7320 (8)	0.5102 (5)	0.2011 (3)	0.0532 (11)	
H12	0.8763	0.5454	0.1787	0.064*	
C13	0.5273 (8)	0.4491 (4)	0.1340 (2)	0.0453 (10)	
C14	0.3274 (8)	0.3949 (5)	0.1727 (3)	0.0607 (13)	
H14	0.1884	0.3480	0.1305	0.073*	
C15	0.3302 (8)	0.4094 (5)	0.2746 (3)	0.0542 (12)	
H15	0.1892	0.3725	0.2984	0.065*	
C16	0.5295 (10)	0.4387 (5)	0.0226 (3)	0.0609 (13)	
H16A	0.6935	0.4220	0.0109	0.073*	
H16B	0.4068	0.3655	−0.0098	0.073*	
O5	0.1581 (5)	0.3715 (3)	0.47022 (17)	0.0317 (6)	
H5A	0.026 (8)	0.368 (4)	0.482 (3)	0.049 (13)*	
H5B	0.216 (8)	0.299 (5)	0.491 (3)	0.057 (14)*	

### Atomic displacement parameters (Å^2^)

**Table:**

	*U*^11^	*U*^22^	*U*^33^	*U*^12^	*U*^13^	*U*^23^
Co1	0.0194 (3)	0.0328 (4)	0.0189 (3)	0.0042 (3)	0.0066 (2)	0.0067 (3)
N1	0.0312 (15)	0.045 (2)	0.0198 (13)	0.0076 (13)	0.0064 (11)	0.0088 (13)
O1	0.0244 (12)	0.0404 (17)	0.0372 (13)	0.0099 (11)	0.0096 (10)	0.0136 (11)
O2	0.0341 (16)	0.046 (2)	0.108 (3)	0.0054 (13)	0.0149 (16)	0.0305 (18)
O3	0.104 (3)	0.095 (3)	0.064 (2)	0.061 (2)	0.026 (2)	0.017 (2)
O4	0.165 (4)	0.123 (4)	0.063 (2)	0.060 (3)	0.036 (3)	−0.007 (2)
C1	0.033 (2)	0.042 (2)	0.0386 (19)	0.0136 (17)	0.0135 (15)	0.0137 (17)
C2	0.049 (2)	0.052 (3)	0.057 (2)	0.021 (2)	0.0152 (19)	0.024 (2)
C3	0.051 (3)	0.056 (3)	0.066 (3)	0.008 (2)	0.010 (2)	0.026 (2)
C4	0.056 (3)	0.050 (3)	0.051 (2)	0.012 (2)	0.002 (2)	0.017 (2)
C5	0.063 (3)	0.060 (3)	0.050 (2)	0.012 (2)	0.007 (2)	0.015 (2)
C6	0.100 (4)	0.089 (5)	0.052 (3)	0.021 (4)	−0.016 (3)	0.019 (3)
C7	0.092 (4)	0.073 (4)	0.083 (4)	0.033 (3)	−0.022 (3)	0.025 (3)
C8	0.073 (3)	0.050 (3)	0.088 (4)	0.022 (3)	−0.006 (3)	0.015 (3)
C9	0.082 (3)	0.045 (3)	0.062 (3)	0.022 (3)	−0.009 (2)	0.003 (2)
C10	0.098 (4)	0.083 (4)	0.056 (3)	0.036 (3)	0.023 (3)	0.016 (3)
C11	0.047 (2)	0.059 (3)	0.0278 (18)	−0.0039 (19)	0.0091 (16)	0.0049 (18)
C12	0.061 (3)	0.067 (3)	0.035 (2)	0.001 (2)	0.023 (2)	0.010 (2)
C13	0.069 (3)	0.052 (3)	0.0201 (17)	0.019 (2)	0.0123 (18)	0.0091 (17)
C14	0.053 (3)	0.097 (4)	0.0250 (19)	0.000 (2)	−0.0016 (18)	−0.007 (2)
C15	0.043 (2)	0.088 (4)	0.0270 (18)	−0.009 (2)	0.0090 (17)	0.002 (2)
C16	0.095 (4)	0.071 (4)	0.0237 (19)	0.033 (3)	0.014 (2)	0.0065 (19)
O5	0.0219 (13)	0.0407 (17)	0.0342 (13)	0.0022 (11)	0.0096 (10)	0.0082 (11)

### Geometric parameters (Å, º)

**Table:**

Co1—O1	2.101 (2)	C5—C10	1.461 (7)
Co1—O1^i^	2.101 (2)	C6—C7	1.362 (7)
Co1—O5^i^	2.108 (3)	C6—H6	0.9300
Co1—O5	2.108 (3)	C7—C8	1.369 (7)
Co1—N1^i^	2.195 (2)	C7—H7	0.9300
Co1—N1	2.195 (2)	C8—C9	1.368 (6)
N1—C15	1.331 (5)	C8—H8	0.9300
N1—C11	1.334 (5)	C9—H9	0.9300
O1—C1	1.266 (4)	C11—C12	1.386 (5)
O2—C1	1.238 (4)	C11—H11	0.9300
O3—C10	1.357 (6)	C12—C13	1.373 (6)
O3—C3	1.464 (6)	C12—H12	0.9300
O4—C10	1.197 (6)	C13—C14	1.360 (6)
C1—C2	1.526 (5)	C13—C16	1.515 (5)
C2—C3	1.488 (5)	C14—C15	1.383 (5)
C2—H2A	0.9700	C14—H14	0.9300
C2—H2B	0.9700	C15—H15	0.9300
C3—C4	1.509 (6)	C16—C16^ii^	1.501 (9)
C3—H3	0.9800	C16—H16A	0.9700
C4—C9	1.379 (6)	C16—H16B	0.9700
C4—C5	1.379 (6)	O5—H5A	0.76 (4)
C5—C6	1.386 (6)	O5—H5B	0.90 (5)
			
O1—Co1—O1^i^	180.000 (1)	C6—C5—C10	131.1 (5)
O1—Co1—O5^i^	89.80 (10)	C7—C6—C5	118.9 (5)
O1^i^—Co1—O5^i^	90.20 (10)	C7—C6—H6	120.5
O1—Co1—O5	90.20 (10)	C5—C6—H6	120.5
O1^i^—Co1—O5	89.80 (10)	C6—C7—C8	120.6 (4)
O5^i^—Co1—O5	180.00 (14)	C6—C7—H7	119.7
O1—Co1—N1^i^	89.22 (9)	C8—C7—H7	119.7
O1^i^—Co1—N1^i^	90.78 (9)	C9—C8—C7	121.6 (5)
O5^i^—Co1—N1^i^	88.53 (10)	C9—C8—H8	119.2
O5—Co1—N1^i^	91.47 (10)	C7—C8—H8	119.2
O1—Co1—N1	90.78 (9)	C8—C9—C4	118.0 (4)
O1^i^—Co1—N1	89.22 (9)	C8—C9—H9	121.0
O5^i^—Co1—N1	91.47 (10)	C4—C9—H9	121.0
O5—Co1—N1	88.53 (10)	O4—C10—O3	121.3 (5)
N1^i^—Co1—N1	180.000 (1)	O4—C10—C5	130.3 (5)
C15—N1—C11	115.3 (3)	O3—C10—C5	108.4 (4)
C15—N1—Co1	121.1 (2)	N1—C11—C12	123.8 (4)
C11—N1—Co1	123.6 (2)	N1—C11—H11	118.1
C1—O1—Co1	128.2 (2)	C12—C11—H11	118.1
C10—O3—C3	110.7 (4)	C13—C12—C11	119.8 (4)
O2—C1—O1	125.1 (3)	C13—C12—H12	120.1
O2—C1—C2	118.2 (4)	C11—C12—H12	120.1
O1—C1—C2	116.7 (3)	C14—C13—C12	116.7 (3)
C3—C2—C1	113.8 (3)	C14—C13—C16	122.0 (4)
C3—C2—H2A	108.8	C12—C13—C16	121.3 (4)
C1—C2—H2A	108.8	C13—C14—C15	120.3 (4)
C3—C2—H2B	108.8	C13—C14—H14	119.9
C1—C2—H2B	108.8	C15—C14—H14	119.9
H2A—C2—H2B	107.7	N1—C15—C14	124.0 (4)
O3—C3—C2	109.7 (4)	N1—C15—H15	118.0
O3—C3—C4	103.6 (3)	C14—C15—H15	118.0
C2—C3—C4	113.4 (4)	C16^ii^—C16—C13	111.5 (4)
O3—C3—H3	110.0	C16^ii^—C16—H16A	109.3
C2—C3—H3	110.0	C13—C16—H16A	109.3
C4—C3—H3	110.0	C16^ii^—C16—H16B	109.3
C9—C4—C5	120.9 (4)	C13—C16—H16B	109.3
C9—C4—C3	131.1 (4)	H16A—C16—H16B	108.0
C5—C4—C3	108.0 (4)	Co1—O5—H5A	138 (3)
C4—C5—C6	119.9 (5)	Co1—O5—H5B	99 (3)
C4—C5—C10	109.0 (4)	H5A—O5—H5B	106 (4)
			
O1—Co1—N1—C15	99.1 (3)	C3—C4—C5—C10	−3.4 (6)
O1^i^—Co1—N1—C15	−80.9 (3)	C4—C5—C6—C7	3.1 (9)
O5^i^—Co1—N1—C15	−171.1 (3)	C10—C5—C6—C7	−177.3 (6)
O5—Co1—N1—C15	8.9 (3)	C5—C6—C7—C8	−0.2 (10)
O1—Co1—N1—C11	−82.1 (3)	C6—C7—C8—C9	−1.4 (10)
O1^i^—Co1—N1—C11	97.9 (3)	C7—C8—C9—C4	0.0 (8)
O5^i^—Co1—N1—C11	7.7 (3)	C5—C4—C9—C8	2.9 (8)
O5—Co1—N1—C11	−172.3 (3)	C3—C4—C9—C8	−178.1 (5)
O5^i^—Co1—O1—C1	161.1 (3)	C3—O3—C10—O4	−176.7 (6)
O5—Co1—O1—C1	−18.9 (3)	C3—O3—C10—C5	2.4 (6)
N1^i^—Co1—O1—C1	72.6 (3)	C4—C5—C10—O4	179.7 (7)
N1—Co1—O1—C1	−107.4 (3)	C6—C5—C10—O4	0.1 (11)
Co1—O1—C1—O2	16.8 (5)	C4—C5—C10—O3	0.7 (6)
Co1—O1—C1—C2	−163.8 (2)	C6—C5—C10—O3	−178.9 (5)
O2—C1—C2—C3	−48.9 (6)	C15—N1—C11—C12	3.5 (6)
O1—C1—C2—C3	131.7 (4)	Co1—N1—C11—C12	−175.4 (3)
C10—O3—C3—C2	−125.7 (4)	N1—C11—C12—C13	−0.9 (7)
C10—O3—C3—C4	−4.3 (5)	C11—C12—C13—C14	−2.9 (7)
C1—C2—C3—O3	−61.7 (5)	C11—C12—C13—C16	179.3 (4)
C1—C2—C3—C4	−177.1 (4)	C12—C13—C14—C15	3.8 (7)
O3—C3—C4—C9	−174.5 (5)	C16—C13—C14—C15	−178.4 (4)
C2—C3—C4—C9	−55.6 (7)	C11—N1—C15—C14	−2.5 (7)
O3—C3—C4—C5	4.6 (5)	Co1—N1—C15—C14	176.4 (4)
C2—C3—C4—C5	123.5 (5)	C13—C14—C15—N1	−1.2 (8)
C9—C4—C5—C6	−4.5 (8)	C14—C13—C16—C16^ii^	100.1 (7)
C3—C4—C5—C6	176.3 (5)	C12—C13—C16—C16^ii^	−82.3 (7)
C9—C4—C5—C10	175.8 (5)		

Symmetry codes: (i) −*x*+1, −*y*+1, −*z*+1; (ii) −*x*+1, −*y*+1, −*z*.

### Hydrogen-bond geometry (Å, º)

**Table:**

*D*—H···*A*	*D*—H	H···*A*	*D*···*A*	*D*—H···*A*
O5—H5*B*···O2	0.90 (5)	1.72 (5)	2.603 (4)	168 (4)
O5—H5*B*···O1	0.90 (5)	2.57 (4)	2.981 (3)	109 (3)
O5—H5*A*···O1^iii^	0.76 (4)	1.99 (4)	2.752 (3)	172 (4)

Symmetry code: (iii) *x*−1, *y*, *z*.
